# Supplementary material for: Parental Vaccine Preferences for Their Children in China: A Discrete Choice Experiment
Source: Vaccines (Basel). 2020 Nov 16;8(4):687. doi: 10.3390/vaccines8040687 (PMC7712304; doi:10.3390/vaccines8040687)
Supplement: Supplementary file 1 [file vaccines-08-00687-s001.pdf]

vaccines

MDPI

Article

Parental vaccine preferences for their children in China: A discrete choice experiment

Tiantian Gong [1,2], Gang Chen [3], Ping Liu[1,2], Xiaozhen Lai[4], Hongguo Rong[5], Xiaochen Ma[5], Zhiyuan Hou[6], Hai Fang [5,7,8,*], Shunping Li [1,2,*]

Supplementary Materials:

Table S1. Mixed logit model results for the severity of diseases coded for dummy variables.

Attributes		SE†	SD	SE‡
Non-vaccination	-2.982***	0.442	4.436***	0.409
Protection rate prevented by a vaccine (ref: 65%)				
80%	0.486***	0.075	0.025	0.147
95%	1.168***	0.085	0.533***	0.135
Risk of severe side effect event (ref: high)				
moderate	0.799***	0.078	0.131	0.269
low	1.662***	0.117	1.398***	0.116
Location of vaccine manufacturer (ref: domestic)				
Imported	-0.154*	0.063	0.741***	0.088
Duration of vaccine-induced protection (ref:1 year)				
5 years	0.296***	0.072	0.031	0.125
10 years	0.598***	0.092	0.881***	0.119
Severity of diseases prevented by vaccines (ref: 1% mortality)				
5% mortality	0.148	0.092	0.093	0.297
10% mortality	0.161	0.090	0.723***	0.142
15% mortality	0.085	0.107	1.263***	0.126
Out-of-pocket Cost	-0.001***	0.000	0.003***	0.000
Log likelihood		-3070.294		
AIC		6188.588		
BIC		6367.635		
Respondents, n		428		
Observations, n		12840		

Note: 1. —coefficient, SE†—standard error of coefficient, SD—standard deviation, SE‡—standard error of SD, ref—reference, AIC—Akaike information criterion, BIC—Bayesian information criterion. All attributes except for cost were coded as dummy variables. 2. A total of 598 parents enrolled in the survey and 580 completed the majority of the questionnaire. Respondents (428) who passed the consistency test were included in the main effects DCE result reported in this table. 3. $*p < 0.05$; $**p < 0.01$; $***p < 0.001$.

Table S2. Conditional logit model results with only the main effects.

Attributes		SE	P-value	95% CI	
Non-vaccination	-0.190	0.154	0.217	-0.490	0.111
Protection rate prevented by a vaccine (ref:65%)					
80%	0.388	0.052	<0.001	0.285	0.490
95%	0.791	0.060	<0.001	0.674	0.908
Risk of severe side effect event (ref: high)					
Moderate	0.530	0.056	<0.001	0.421	0.640
Low	1.029	0.075	<0.001	0.881	1.177
Location of vaccine manufacturer (ref: domestic)					
Imported	-0.122	0.043	0.004	-0.206	-0.038

Duration of vaccine-induced protection (ref:1 year)					
5 years	0.163	0.048	0.001	0.069	0.256
10 years	0.397	0.065	<0.001	0.270	0.524
Out-of-pocket Cost	-0.001	0.000	0.003	-0.002	-0.001
Severity of diseases prevented by vaccines (per 1%)	0.006	0.007	0.353	-0.007	0.019
Log likelihood			-3800.979		
AIC			7621.958		
BIC			7696.561		
Respondents, n			428		
Observations, n			12840		

Note: 1. CI—confidence interval. All attributes except for cost and severity of diseases prevented by vaccines were coded as dummy variables. 2. A total of 598 parents enrolled in the survey and 580 completed the majority of the questionnaire. Respondents (428) who passed the consistency test were included in the main effects DCE result reported in this table.

Table S3. Mixed logit model results for the full sample

**Attributes**		**SE**	**SD**	**SE**
Non-vaccination	-2.397***	0.285	3.861***	0.266
Protection rate prevented by a vaccine (ref: 65%)				
80%	0.558***	0.060	0.008	0.130
95%	1.090***	0.069	0.618***	0.097
Risk of severe side effect event (ref: high)				
moderate	0.652***	0.061	0.070	0.214
low	1.386***	0.087	1.267***	0.090
Location of vaccine manufacturer (ref: domestic)				
Imported	-0.122**	0.043	0.659***	0.071
Duration of vaccine-induced protection (ref: 1 year)				
5 years	0.229***	0.058	0.015	0.123
10 years	0.507***	0.070	0.731***	0.093
Out-of-pocket Cost	-0.001***	0.000	0.003***	0.000
Severity of diseases prevented by vaccines (per 1%)	0.014	0.008	0.140***	0.010
Log likelihood		-4414.636		
AIC		8869.272		
BIC		9024.557		
Respondents, n		580		
Observations, n		17400		

Note: 1. All attributes except for cost and severity of diseases prevented by vaccines were coded as dummy variables. 2. A total of 598 parents enrolled in the survey. 580 parents completed the majority of the questionnaire and were included in the main effects DCE result reported in this table. 3. *$p < 0.05$; **$p < 0.01$; ***$p < 0.001$.
